# A Diagnosis

**Published:** 1886-09-20

**Authors:** H. Christopher

**Affiliations:** St. Joseph, Missouri


### A DIAGNOSIS.

BY H. CHRISTOPHER, M.D., OF MISSOURI.

The following case is one of interest in that one morbid action simulates that of another of quite a different kind, and one that makes it important that a mistake should not be made in diagnosis. The patient, a physician, may tell his own story, as I should but repeat his words were I to attempt a description from the details he gave me.

"In June, 1877, I attended a convention in the proceedings of which I was much interested. I was in good health, or, at least, in usual health. I had never up to that time had any trouble in any part of the urinary apparatus. Micturition was as free and easy as at any time during my life.

"A collation was spread in the shade of the house and surrounding trees, and was partaken of, standing, from high tables. I was under a pleasurable excitement, and felt as well as any one in perfect health. I had, however, for many years suffered at times from hepatic indigestion. Was never ailing since maturity from any other cause. On the morning, however, of the second day of the convention, I awoke with the first and severest attack of palpitation that I have ever experienced. I could find no relief in any position. Until breakfast (some two hours) the palpitation continued, but gradually less violently. Just as we went into breakfast I took about an ounce of brandy; ate a good breakfast, and felt no more palpitation. During the day, however, when a desire to micturate became pressing, I sought relief in voiding it; but was greatly surprised to find that the urine would not flow except slowly and at intervals. I was still under considerable mental excitement, and did not, at one time, I suppose empty the bladder. This difficulty in voiding urine continued for several days after I returned home, and has not entirely left me since. It occurs, however, only when micturition is postponed too long, as during sleep, and on sleeping very profoundly, even though the sleep be but for a short time. On such occasions micturition is slow and somewhat painful. When not allowed to accumulate, the urine is voided without difficulty or pain. But the evacuation may be difficult and painful and free and easy all within twelve hours or less.

"The difficulty occurred at the beginning. When once it starts the fluid flows pretty freely, but with some tenesmic pain along the urethra; but if the pain at the beginning be much, the difficulty is prolonged. The pain extends from the cirvix to the fossa navicularis, and is of a tenesmic aching character and continues after evacuation.

"I first thought the cause might be a partial paresis of the muscles of the cyst resulting from distension, and I took nux vomica, but with no benefit. I then concluded that the trouble lay in the prostate, and hence discontinued remedies. The same trouble has now continued for nine years better and worse at times.

"On the 22nd of last month (June) I again attended a convention. Here I was more than usually careful, as respects eating and mental excitement, and gave attention to the bladder as well as circumstances would permit; but as the event proved, the attention was not sufficient.

"At 5 p. m., I found that I could not void the urine, though the desire was great; and having but little time to wait on the action of the muscles concerned in micturition, I had it drawn. The catheter was introduced without the slightest difficulty, and some twelve ounces were voided. About 5 p. m., I passed with slight pain or trouble what had accumulated in the hour. Some little tenesmic pain followed the evacuation.

"The physician draining the urine attributed the whole trouble to an enlarged prostate; but I was not of his opinion. He had not heard the history of the case. I had formed no satisfactory opinion of the case.

"During the next day I was careful to keep the cyst empty. There was no trouble attending the evacuation of the urine but what arose from the tenesmic feeling accompanying and following micturition, a pain extending from the cervix to the glans, and continuing more or less during the intervals. On the night of the 23rd I had a rigor and a little nausea. After this the pain along the urethra was constant and extended to the rectum and sphincter ani, accompanied by a feeling as though the rectum were full and needed emptying. The pains in the urethra was of an aching and tenesmic character; that of the rectum and the sphincter ani, aching. The 24th found me no better; and at 10 a. m. I went to bed and had a severe rigor, followed by vomiting. There had been no digestion for more than twelve hours at least. The pain in the urethra and rectum and sphincter continued up to the time of vomiting, when it ceased, and was felt little afterward. I took about 6 grains of calomel at night in powder, and the same next morning in pills; and though it produced no motion of the bowels, I felt but little pain in the urethra on the 25th. I had some fever on the 24th and 25th. On the night of the 25th I took about the same amount of calomel, and again on the night of the 26th the same. All of the 26th I had lumbago so severely that I could scarcely get about. I took another dose of calomel at night, and on the 28th 'all bands were loosed,' and I felt that 'Richard was himself again'

"Not eating prudently during the next few days, I awoke at 4 a. m., on the morning of the 3th inst. to void urine which was attended with like difficulty and pain. When I lay down again a pain suddenly seized me just to the left the median line and on a line with the cervix vesical, which continued to increase in intensity, and extended to a point just opposite in the lumbar region. The distress became so great that respiration was difficult. Believing that I was in for at attack similar to one that I had twenty years before, I awoke my wife and called for the calomel vial. I poured out about 6 or 8 grains and took it, with the expectation that it would give me relief in about an hour, môre certainly than any anodyne would. For an hour I suffered from the pain in hypogastric and lumbar regions until I became extremely weak. I endured it, however, as well as I could, when, at the end of an hour, the pain left me as suddenly as it seized me. I then called to mind the circumstances of an attack I had of the same kind in October, 1861. Since that time I have treated many attacks like that in the loins, which I have always diagnosed as muscular rheumatism.

"Now, I will tell you what *my* diagnosis is in respect to the trouble in micturition as I have just described it. I am fully satisfied that the trouble arises from rheumatism of the muscles concerned in the act of micturition, except those of the body of the cyst, the muscles of the cervix, prostate, corpus cavernosum and those of the urethra; and I am led to this conclusion by several considerations: First, the character of the pain, the occurrence of a like pain in the muscles of the rectum, sphincter ani, and those of the abdominal walls, anterior and posterior, as occurred in the attack of muscular rheumatism of the 4th inst. I remember that in the attack I had in 1861 that it shifted finally from the muscles of the right illiac to those of the cervix and produced strangury. In the second place, the same remedy relieved the pain and difficulty of micturition felt in the whole length of the urethra, viz: calomel. I have never known calomel to fail in relieving, in very short time, the pain of muscular rheumatism, and I have treated a large number of cases, both acute and chronic. This is my diagnosis, and this my uniform treatment for this form of rheumatism."

I submit this case as presenting something new, and, as I loôk at it, important. I note that the party, now in his sixty-seventh year, was at the time of the first appearace of the trouble in voiding urine, fifty-eight years of age, and had never had up to that time any symptoms of an enlarged prostate. It could not therefore, have been the cause, since, in addition to this fact, the attack of dysuria was too sudden. Its connection with muscular rhumatism in adjacent muscles and its relief by calomel are condusive reasons for diagnosing such a case as this as muscular rheumatism. I am satisfied that the medical patient is correct in this view of the case. It is, therefore, important that the discrimination should be made between enlarged prostate and simple rheumatism of the muscles concerned in the act of micturition. A patient can be saved from much pain by recognizing the true nature of the disease, and treating it as a functional derangement, and not as an incurable disease—an enlarged prostate. This form of rheumatism is always the result of hepatic disturbance, and can, when acute, always be quickly relieved by the mild chloride in full doses; and, when chronic, by the same remedy, continued in small doses until the cure comes, as come it will. A large experience with this remedy in this affection enables me to speak with confidence. But this is the first case that I have ever known where the rheutism has seized on the muscles concerned in micturition, producing symptoms that might easily be mistaken for those of an enlarged prostate. I submit the case as one new to me, and possibly to others.

*St. Joseph, Mo, July 24, 1886.*

